# Underexplored moderating effects of sex in subjective cognitive decline: A systematic review and evidence gap

**DOI:** 10.1177/13872877251397411

**Published:** 2025-11-28

**Authors:** Yolanda Álvarez-Pérez, Andrea Duarte-Díaz, Yaiza Molina, Nerea Figueroa-Lamas, Patricia Diaz-Galvan, Eloy García-Cabello, Lissett Gonzalez-Burgos, Roraima Yánes-Pérez, Amado Rivero-Santana, Jonas K Olofsson, Jose Barroso, Daniel Ferreira, Lilisbeth Perestelo-Pérez, Nira Cedres

**Affiliations:** 1 Canary Islands Health Research Institute Foundation (FIISC), El Rosario, Spain; 2 Evaluation Unit (SESCS), Canary Islands Health Service (SCS), El Rosario, Spain; 3 Network for Research on Chronicity, Primary Care and Health Promotion (RICAPPS), Madrid, Spain; 4 Faculty of Health Sciences, University Fernando Pessoa Canarias, Las Palmas de Gran Canaria, Spain; 5 Faculty of Psychology, University of La Laguna, San Cristobal de La Laguna, Spain; 6 Instituto de Biomedicina de Sevilla (iBIS), Sevilla, Spain; 7 Department of Psychology, Sensory Cognitive Interaction Laboratory (SCI-lab), Stockholm University, Stockholm, Sweden; 8 Division of Clinical Geriatrics, Centre for Alzheimer Research, Department of Neurobiology, Care Sciences, and Society, Karolinska Institutet, Stockholm, Sweden; 9 Theme Inflammation and Aging, Karolinska University Hospital, Stockholm, Sweden

**Keywords:** Alzheimer's disease, dementia, gender, precision medicine, subjective cognitive impairment

## Abstract

**Background:**

Subjective cognitive decline (SCD) is considered an early symptom of Alzheimer's disease (AD) and other types of dementia. Establishing differences between males and females in the presentation and risk factors associated with SCD is critical for utilizing subjective cognitive assessments in prognosticating dementia.

**Objective:**

We performed a comprehensive review of studies examining the moderating effect of sex on the association between SCD and relevant physical and/or mental health-related outcomes.

**Methods:**

This study was performed following the PRISMA guidelines. We conducted database search in Medline, Cochrane, Web of Science (WOS), and CINAHL. Primary studies including data of the moderating effect of sex on the association between SCD and different outcomes were selected.

**Results:**

A total of 16 studies were included. We found limited evidence for a moderating effect of sex in SCD. Most of the available literature explored sex differences in SCD for risk of dementia, cognitive performance, competing risk of death, AD biomarkers, basal forebrain resting-state functional connectivity, brain volume, among other health outcomes. Among SCD individuals, females showed increased risk of cognitive decline, dementia and other health outcomes, whereas males showed increased risk of death and longer sickness absence compared to controls.

**Conclusions:**

Our comprehensive review denotes a lack of studies directly testing the moderating effect of sex in SCD. The available literature points to sex specific associations between SCD and multiple clinical outcomes. However, in line with the current effort of the SCD initiative, further research is necessary within this emerging topic.

## Introduction

Subjective cognitive decline (SCD) has been proposed as an early indicator of Alzheimer's disease (AD), potentially reflecting the preclinical stage before objective cognitive changes are detectable.^1^ Longitudinal studies have demonstrated that up to 27% of individuals with SCD develop cognitive impairment and clinically progress to mild cognitive impairment or dementia.^2,3^ Following the publication of a conceptual research framework for SCD,^4^ there is a stronger focus on the characteristics and risk factors that could explain or moderate the progression from SCD to clinical impairment.^1^ The prevalence of AD biomarkers during the SCD stage is up to 35.9% for amyloid, 33.6% for phosphorylated tau (p-tau) and 46.3% for total tau.^5^ Interestingly, results show that SCD not only increases the risks for developing AD but also other dementias.^3^ In fact, cerebrovascular disease is often found in SCD compared to controls.^6^ This evidence underscores the possible heterogeneity within the SCD population in terms of underlying pathology.

The scientific literature points to specific contributions of sex to this heterogeneity, in terms of risk and clinical presentation. Females comprise roughly two-thirds of AD cases,^7^ whereas males show increased risk of developing vascular dementia in the presence of cardiovascular risk factors.^8^ Biological factors, such as hormonal changes or *APOE* ε4 carriership, and sociocultural influences, including help-seeking behavior or cognitive reserve, may contribute to these observed sex disparities.^7,9,10^ For instance, the transition to menopause has been associated with accelerated brain aging and amyloid accumulation,^10,11^ whereas men may underreport early cognitive symptoms due to cultural norms around health-seeking.^12,13^ Clarifying whether sex-specific mechanisms moderate the association of SCD with different clinical outcomes is therefore critical for identifying those most at risk of progression.

Although by definition SCD individuals do not present with cognitive impairment, subclinical variability in cognition have been described.^14^ Poorer cognitive performance within normal thresholds in processing speed, executive functions, memory, and visuospatial abilities have been described in older individuals with SCD.^15,16^ Other important findings in SCD individuals are self-perceived changes in functionality,^14^ and increased depressive and anxiety symptoms.^17–20^ Importantly, one of the key debates in the field is whether subjective complaints reflect mood states rather than neurodegenerative processes. However, it has been demonstrated that the association of SCD and neurodegeneration biomarkers is independent of depressive symptomatology.^17,20^ Thus, self-reported changes in cognition, functionality and mood are also frequent in SCD. Understanding whether SCD associations vary for males and females could help disentangle the variability observed at this earliest stage.

This evidence indicates that SCD, as originally defined by the SCD-I, can be associated with multiple clinical outcomes, including risk of dementia,^3,5^ variability in cognition,^16,17^ functionality,^14^ biomarkers^21,22^ and mental health.^23,24^ To advance in precision medicine and to unravel clinical heterogeneity in SCD individuals, it is necessary to better understand the factors that may influence or moderate the associations of cognitive complaints with relevant clinical symptoms. The recent literature review from the SCD-I summarizing the latest contributions to the knowledge about SCD points to disentangling such heterogeneity as a priority of the future research in the field.^25^ As a result, the SCD-I has included the category of SCD-plus as a status of increased risk characterized by memory-specific complaints, onset within the last five years, concern associated with decline, and age >60. However, whether sex contributes to increased risk is yet to be determined. Generating a clearer picture of sex-specific SCD patterns will be invaluable for refining risk stratification, defining preventive approaches, and advancing precision medicine in the field of neurodegenerative diseases. Accumulating research suggests that there may be differences in the prevalence, presentation, and factors associated with SCD between males and females.^9^ Hormonal factors, genetics, and underlying pathologies have been postulated as potential causes of these differences.^9^ However, whether these sex differences moderate the association between SCD and different outcomes is still poorly understood. Therefore, the aim of this study is to investigate the moderating effect of sex on the association between SCD and relevant physical and/or mental health-related outcomes by means of a comprehensive narrative synthesis of empirical studies.

## Methods

The review protocol for this study was prospectively registered in PROSPERO (CRD42023392799) and followed the Preferred Reporting Items for Systematic Reviews and Meta-Analyses (PRISMA) statement.^26^

The current study is part of a larger project based on a single registration and search strategy to evaluate the moderating effect of sex, age, and education on the association between SCD and relevant outcome measures. The analysis of age and education are beyond the scope of the present work and will be presented separately; while, the joint systematic review protocol can be consulted here https://www.crd.york.ac.uk/prospero/display_record.php?RecordID = 392799.

### Search strategy and study selection process

To identify relevant studies, we conducted a comprehensive search of multiple databases, including Medline, Cochrane, Web of Science (WOS), and CINAHL from their inception dates to February 2022. Our search strategy utilized additional terms related to SCD (i.e., “*subjective cognitive complaint” OR “cognitive complaint” OR “subjective cognitive impairment” OR “subjective memory decline” OR “subjective memory complaint” OR “memory complaint” OR “subjective memory impairment”*) and sex (i.e., “*gender” OR “sex” OR “women” OR “men” OR “male” OR “female”*) (Supplemental Table 1). As we acknowledge that sex and gender are often used interchangeably in the scientific literature, we have therefore used both concepts in our search to maximize coverage of the scientific literature. In our reporting, we will use the terms “sex” and “female/male” because of the biological nature of our research,^27,28^ but it should be noted that the classification is based on self-reports. No restriction on language was imposed in the initial search.

Two reviewers independently screened titles and abstracts for relevance to the selection criteria, with any disagreements resolved through discussion or with the participation of a third reviewer. The inclusion criteria were applied to the full-text articles deemed relevant, which were then further evaluated and selected for inclusion in the study.

### Selection criteria

We used the following inclusion criteria for our study:

- Studies where there was a study group with SCD, as defined by Jessen research criteria,^1^ which include 1) self-experienced persistent decline in cognitive capacity in comparison with a previously normal status and unrelated to an acute event, and 2) normal age-, sex-, and education-adjusted performance on standardized cognitive tests used to classify mild cognitive impairment (MCI) or prodromal AD. Studies operationalizing SCD as subjective memory decline, subjective cognitive impairment, subjective memory impairment, subjective cognitive complaints, or subjective memory complaints were considered.
- Studies that assessed the moderator effect of sex in the association of SCD with any relevant physical and/or mental health-related outcome (e.g., progression to dementia, depression, physical activity). If no studies directly analyzing the moderator effect were found, studies where the moderating effect could be indirectly assessed were also included (i.e., comparing groups based on sex).
- Articles written in English or Spanish.

We excluded studies with patients diagnosed with MCI/prodromal AD or dementia. Additionally, we excluded studies where SCD could be explained by a psychiatric or neurological disease, medical disorder, medication usage, or substance use. Studies where it was not possible to extract the moderator effect between sex and any physical and/or mental health-related outcome due to the type of analysis (e.g., adjusted models) were also excluded.

### Data extraction and quality assessment

Two reviewers independently extracted and evaluated study characteristics and assessed the risk of bias. In case of any discrepancies, a third review author was consulted, and discussions were held to achieve a consensus. Data extraction was conducted using a spreadsheet file that captured relevant information from each study. This file included data collection dates, sample characteristics (age, sex, diagnosis), study design, type of SCD measure (validated questionnaires, etc.), SCD domains (memory, attention, language, etc.), and outcomes measures (progression to dementia, depression, biomarkers, etc.).

To assess the methodological quality of observational studies, we used Joanna Briggs Institute (JBI) critical appraisal tools.^29^

### Data synthesis

We conducted a comprehensive narrative synthesis, which involved summarizing the findings in a descriptive and qualitative manner. Due to substantial heterogeneity among the studies and the limited number of studies available for each outcome measure, a meta-analysis could not be performed. The included studies varied in their analytical approaches, with some examining sex differences while others tested interactions, further complicating the comparability of findings. To facilitate the analysis, we grouped the studies based the reported outcomes. By comparing and contrasting the findings across studies, we identified similarities and differences. Subsequently, we drew conclusions based on this narrative synthesis approach. By adopting this methodology, we aimed to provide a thorough and comprehensive summary of the evidence while recognizing and addressing the heterogeneity observed across the included studies.

## Results

**Figure 1.**
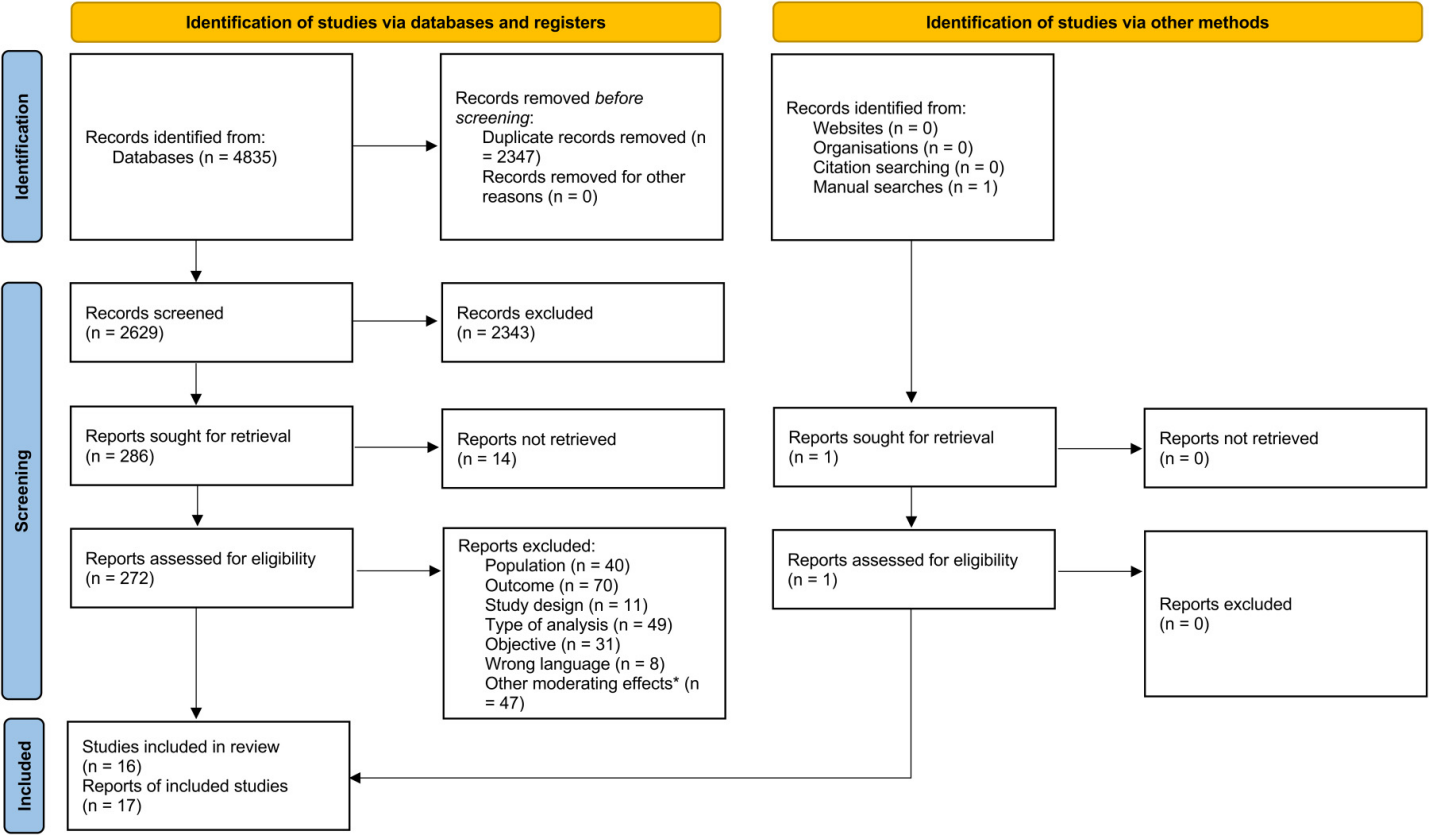
PRISMA flow diagram.

The initial search in the electronic databases yielded 4835 references. After removing duplicates and screening by title and abstract, 272 full-text articles were assessed for eligibility. In addition, one relevant study not retrieved in the database search was identified through manual searches. Consequently, 16 studies (17 references)^30–46^ were finally included (Figure 1). Of the 16 studies analyzed, 6 directly explored the moderating effect of sex in the association of SCD with at least one health-related outcome^33–36^^,41,44^ while 10 relied on group comparisons or other statistical approaches rather than formal interaction analyses.^30–32^^,36–43,45,46^ Because the latter did not directly test for moderation, they were included in the systematic review only to explore sex differences that might indirectly suggest a moderating effect.

### Characteristics of the included studies

All 16 studies had an observational design (7 cross-sectional and 9 cohort studies). Although sex and gender are often used interchangeably in the scientific literature, the concepts are not interchangeable. However, self-reported gender as a proxy of sex was used to operationalize sex variable for all the studies included. The variables evaluated as associated health outcomes in SCD were the risk of dementia, risk of death, dementia biomarkers, urinary cortisol level, basal forebrain resting-state functional connectivity (RSFC), cognitive performance, frailty, mobility and balance, sickness absence, work disability, and mental health. The characteristics of the included studies are provided in Table 1.

**Table 1.** Characteristics of the included studies.

Author, country (year)	Design	n SCD (% females)	Mean age SCD	Education level	Cognitive domain	Outcomes measures
Abdulrahman (2022)^30^ Netherlands	Observational (prospective)	Total sample: 3409 (54.4) Isolated SCM: 250 (NI) SMC + IADL-I: 351 (NI)	Total sample: 74.4 (2.5) Isolated SCM: NI SMC + IADL-I: NI	Total sample: <7 years: 817 (24.2%) 7–12 years: 1915 (56.7%) >12 years: 643 (19.1%) Isolated SCM: NI SMC + IADL-I: NI	**Subjective Memory Complaints** (item 10 of the 15-item Geriatric Depression Scale: “*Do you feel you have more problems with memory than most?”*)	Risk of dementia Mortality Dementia-mortality
Babapour (2020)^31^ Netherlands	Observational (cross-sectional)	427 (39)	Females: 28 (1.6) Males: 28 (1.8)	Females: 1032 (252) Males: 1070 (263)	Subjects were labeled as having SCD when results of clinical examinations and test results were normal (i.e., criteria for MCI or dementia were not fulfilled, and no psychiatric diagnosis was given).	Aβ42 t-TAU p-TAU
Chiesa (2019)^32^ France	Observational (retrospective)	267 (63)	75.8 (3.5)	NI	**Subjective Memory Complaints** (two items: “Are you complaining about your memory?”, and “Is it a regular complaint that has lasted now more than 6 months?”	Basal Forebrain RSFC–SUVR Association
Cipolli (1990)^33^ Italy	Observational (cross-sectional)	180 (56.67)	65.7 (NI)	NI	**Subjective Memory Complaints** (*Sehulster Memory Scale*)	Randt Memory Test: acquisition-recall and delayed memory.
Drouin (2020)^34^ Canada	Observational (prospective)	580 (65)	70.2 (8.60)	15.3 (3.0) (years)	**Subjective Memory Complaints** (*The Metamemory in Adulthood* and *The Memory Compensation Questionnaire*)	Episodic Memory (the Victoria Longitudinal Study)
Heser (2019)^35^ Germany	Observational (prospective)	1393 (62)	NI	NI	**Memory:** standardized answers to the question: “Do you feel like your memory is178becoming worse?”	Risk of dementia
Kryscio (2014)^36^ USA	Observational (prospective)	296 (NI)	81.5	NI	**Subjective Memory Complaints**	Transition to dementia Risk of death
Oliver (2022)^46^ USA	Observational (prospective)	831 (76)	NI	NI	**Subjective Memory Complaints**	Cognitive performance (global cognition, episodic memory, semantic memory, processing speed, visuospatial ability, working memory)
Peres (2011)^37^ France	Observational (prospective)	1788 (62.3)	NI	NI	**Subjective Memory Complaints:** 1) Forgetfulness in daily living activities; 2) difficulties in retrieving or remembering new information; and 3) difficulties in retrieving or remembering old memories.	Risk of dementia over 15 years of follow-up Risk of dementia at more or less long time
Pihlajamaki (2020)^38^ Finland	Observational (prospective)	Abnormal SCC: 1058 (45.5)	NI	NI	Subjective Cognitive Decline: Memory difficulties; Difficulties in planning and organizing own work tasks; Forgetting agreed issues and work tasks; Difficulties in concentration; Delays in recollection; Disruptions to thinking; Difficulties in recollection	Sickness absence
Pihlajamaki (2021)^39^ Finland	Observational (prospective)	7161 (46)	46.8	N.I.	Subjective Cognitive Decline: Memory difficulties; Difficulties in planning and organizing own work tasks; Forgetting agreed issues and work tasks; Difficulties in concentration; Delays in recollection; Disruptions to thinking; Difficulties in recollection	Work disability
Snitz (2015)^40^ USA	Observational (cross-sectional)	92 (48.9)	81.2	15.4 years (means)	Memory function Cognitive failures Subjective Cognitive Complaints	Brain amyloid-β deposition
Strand (2018)^41^ Norway	Observational (prospective)	368 (48.6)	N.I.	42% ≥ 13 years education	Cognitive function Function in activities of daily living	Relative survival
Wang (2018)^43^ USA	Observational (cross-sectional)	69 (57.97)	71.65	16 years (mean)	Memory	Neuropsychological Assessment: Global cognitive function Memory Attention/executive function Language Visuospatial Depression Functional Neuropsychiatric Amyloid accumulation Biomarkers: Volumes of hippocampus, entorhinal cortex, fusiform and medial temporal lobe and cerebrospinal fluid
Wolf (2005)^44^ USA	Observational (cross-sectional)	27 (59.26)	62.86	16.27 years (mean)	Memory	Urinary cortisol level
Yoon (2020)^45^ Korea	Observational (cross-sectional)	96 (56.3)	N.I.	8.8 years (mean)	Memory	Mobility Balance
Zhang (2018)^47^ USA / Canada	Observational (cross-sectional)	99 (57.6)	71.3	17 years (mean)	Memory Cognitive	Amyloid positivity

IADL-I: Instrumental Activities of Daily Living; MCI: mild cognitive impairment; N.I.: not information; RSFC–SUVR: Resting-state functional connectivity-Standardized uptake value ratio; SCD: Subjective cognitive decline; SMC: Subjective memory complaints

### Risk of bias in the included studies

The primary sources of bias in the cross-sectional studies were related to the criteria used to operationalize SCD and the identification and evaluation of confounding factors. In cohort studies, the main risk of bias was related to possible losses to follow-up and strategies for addressing incomplete follow-up. A detailed quality assessment of each study can be found in Table 2 and Table 3.

**Table 2.** Checklist for quality assessment in cross sectional studies (JBIEE).

Author (year)	1. Were the criteria for inclusion in the sample clearly defined?	2. Were the study subjects and the setting described in detail?	3. Was the exposure measured in a valid and reliable way?	4. Were objective, standard criteria used for measurement of the condition?	5. Were confounding factors identified?	6. Were strategies to deal with confounding factors stated?	7. Were the outcomes measured in a valid and reliable way?	8. Was appropriate statistical analysis used?
Babapour (2020)^31^ Netherlands	Yes	Yes	Yes	Yes	Yes	Yes	Yes	Yes
Cipolli (1990)^33^ Italy	Yes	Yes	Yes	Yes	Yes	Yes	Yes	Yes
Snitz (2015)^40^ USA	Yes	Yes	Yes	Unclear	Yes	Yes	Yes	Yes
Wang (2018)^43^ USA	Yes	Yes	Yes	Yes	Unclear	Unclear	Yes	Yes
Wolf (2005)^44^ USA	Yes	Yes	Yes	Yes	Unclear	Unclear	Yes	Yes
Yoon (2020)^45^ Korea	Yes	Yes	Yes	Yes	Yes	Yes	Yes	Yes
Zhang (2018)^47^ USA / Canada	Yes	Yes	Yes	Unclear	Yes	Yes	Yes	Yes

**Table 3.** Checklist for quality assessment in Cohort studies (JBI).

Author (year)	1. Were the two groups similar and recruited from the same population?	2. Were the exposures measured similarly to assign people to both exposed and unexposed groups?	3. Was the exposure measured in a valid and reliable way?	4. Were confounding factors identified?	5. Were strategies to deal with confounding factors stated?	6. Were the groups/participants free of the outcome at the start of the study (or at the moment of exposure)?	7. Were the outcomes measured in a valid and reliable way?	8. Was the follow up time reported and sufficient to be long enough for outcomes to occur?	9. Was follow up complete, and if not, were the reasons to loss to follow up described and explored?	10. Were strategies to address incomplete follow up utilized?	11. Was appropriate statistical analysis used?
Abdulrahman (2022)^30^ Netherlands	Yes	Yes	Yes	Yes	Yes	Yes	Yes	Yes	Unclear	Unclear	Yes
Chiesa (2019)^32^ France	Yes	Yes	Yes	Yes	Yes	Yes	Yes	Yes	Unclear	Unclear	Yes
Drouin (2020)^34^ Canada	Yes	Yes	Yes	No	No	Yes	Yes	Yes	No	Yes	Yes
Heser (2019)^35^ Germany	Yes	Yes	Unclear	Yes	Yes	Yes	Yes	Yes	Unclear	Unclear	Yes
Kryscio (2014)^36^ USA	Yes	Yes	Yes	No	No	Yes	Yes	Yes	Unclear	Unclear	Yes
Oliver (2022)^46^ USA	Yes	Yes	Yes	Yes	Yes	Yes	Yes	Yes	Yes	Yes	Yes
Peres (2011)^37^ France	Yes	Yes	Yes	Yes	Yes	Yes	Yes	Yes	Yes	Yes	Yes
Pihlajamaki (2020) ^38^ Finland	Yes	Yes	Yes	Yes	Yes	Yes	Yes	Yes	Yes	Yes	Yes
Pihlajamaki (2021)^39^ Finland	Yes	Yes	Yes	Yes	Yes	Yes	Yes	Yes	Yes	Yes	Yes
Strand (2018)^41^ Norway	Yes	Yes	Yes	Yes	Yes	Yes	Yes	Yes	Yes	Yes	Yes

### The moderating effect of sex

#### Risk of dementia

We found two studies^37^ directly testing for the moderating effect of sex on the association between SCD and risk of dementia, and two studies^30,36^ evaluating sex differences on risk of dementia in people with SCD.

Pérès et al. (2011)^37^ observed that, after controlling for educational level, marital status, depressive symptomatology, and Mini-Mental State Examination (MMSE) score, SCD was independently associated with greater risk of dementia over 15 years of follow-up (HR = 1.50; CI = 1.25–1.79). A significant interaction by sex was found, with females having higher risk of dementia (HR = 1.88; 95% CI: 1.48–2.41) than males (HR = 0.99; IC 95%: 0.74–1.33) (p-interaction = 0.002).

Similarly, Heser et al. (2019)^35^ reported that, in models adjusted for age, education, cognitive status, and depressive symptoms, SCD at baseline increased the risk of subsequent dementia (p < 0.001), and this effect was significantly less pronounced in males (interaction: p = 0.022). Stratified analyses showed that SCD increased the risk for subsequent dementia in females (HR = 1.77, p < 0.001), but not in males (HR = 1.07, p = 0.682).

Abdulrahman et al. (2022)^30^ investigated how sex affects the association between SCD (operationalized based on subjective memory complaints) and the risk of developing dementia. Individuals with SCD had an 85% higher dementia risk (HR = 1.85, 95% CI = 1.17–2.90, p = 0.008) compared to individuals without SCD. However, there was no evidence of sex-related differences, as the difference in HRs for males and females was not significant (p-interaction = 0.57). Interestingly, when individuals presented SCD jointly with complaints about their functionality (i.e., Instrumental activities of Daily Living), females showed significantly higher risk of progression to dementia (HR =2.85, 95% CI = 1.65–4.91; p < 0.001).

Kryscio et al. (2014)^36^ evaluated sex differences on risk of transition to cognitive impairment, dementia versus transition to death in people with SCD. They found that SCD females had higher risk of progressing to dementia (OR = 2.6, 95%CI = 1.1–5.8).

The available studies consistently suggest that females with SCD are at higher risk of developing dementia than men, although findings are mixed when considering additional factors such as functional complaints or worries.

#### Cognitive performance

We found three studies exploring a moderating effect of sex in the association of SCD with subclinical variability in cognitive performance,^33,34,46^ and one study exploring sex-differences within SCD.^43^ All four studies focused only on the memory domain, except Wang et al., (2018)^43^ which also investigated global cognition.

Using a longitudinal approach, Drouin et al. (2020)^34^ showed that higher memory complaints at baseline predicted lower memory performance in the next 4.5 years for both males and females. However, baseline SCD based on memory complaints was associated with steeper memory decline in SCD females compared to males.

Cipolli et al. (1990)^33^ studied the moderating effect of sex in the association of SCD operationalized based on memory complaints and the acquisition-recall and delayed recall of new verbal information. They did not find statistically significant moderating effect of sex for the association between memory complaints and the studied memory subcomponents.

Oliver et al. (2022),^46^ in a sample of 3019 cognitively unimpaired older adults, examined sex differences in cognitive trajectories among individuals with SCD. They found that baseline performance varied by sex, with males showing significantly lower scores in global cognition, episodic memory, and perceptual speed, but higher scores in visuospatial abilities compared to females. Moreover, a significant three-way interaction between sex, SCD classification, and time indicated that females with SCD experienced a faster decline than males with SCD across all cognitive domains (global cognition, episodic memory, semantic memory, processing speed, visuospatial ability, and working memory).

Wang et al. (2018)^43^ showed that, within SCD individuals, females showed significantly better performance in different memory tasks, including the Rey Auditory Verbal Learning Test-5 (RAVLT-5) total sum and immediate recall, compared with SCD males. Furthermore, they also found that SCD females performed significantly better in global cognition compared to SCD males operationalized by the Assessment Scale-Cognitive subscale 13 (ADAS-13).

Overall, the evidence indicates that although baseline memory complaints predict future decline in both sexes, females with SCD present a steeper decline over time, while cross-sectional comparisons often reveal equal or even superior cognitive performance in females compared to men.

#### Competing risk of death

We did not find any study directly testing for the moderating effect of sex on the association between SCD and competing risk of death. Nonetheless, three studies evaluated sex differences in mortality among people with SCD.

Abdulrahman and colleagues (2022)^30^ found no difference in risk of mortality between individuals with SCD compared to those without SCD (p = 0.29). Within the SCD group, they also found no evidence of a sex effect in the risk of death (p = 0.76). To note, when individuals presented SCD jointly with subclinical complaints about their functionality (i.e., Instrumental activities of Daily Living), males showed significantly higher risk of death (HR = 1.59, 95% CI = 1.07–2.37; p = 0.02).

In the study by Strand and colleagues (2018)^41^ among SCD patients, the median survival time at age 75 years was 9.2 years (95% CI; 7.5–10.8) for males and 12.0 years (95% CI; 9.1–15.0) for females. Thus, SCD males survived a median of 3 years less than SCD females, however, the analyses were not adjusted for confounders. To note, the survival of the SCD individuals was comparable to that in the general population.

In the study by Kryscio and colleagues (2014),^36^the authors found no significant effect of sex in SCD individuals’ risk of death.

Overall, males with SCD generally showed shorter survival times and increased risk of death compared to females, particularly when SCD co-occurred with functional complaints.

### Alzheimer's disease biomarkers

We did not find any study directly testing for the moderating effect of sex on the association between SCD and AD biomarkers. Nonetheless, four studies evaluated sex differences on AD biomarkers in people with SCD.^31,40,43,47^ All four investigated amyloid-beta biomarkers and two investigated tau biomarkers of AD.^31,43^

Babapour and colleagues (2020)^31^ showed that, within SCD individuals, females had more tau-related pathology as assessed through cerebrospinal fluid (CSF) biomarkers, compared with males. However, these differences were only significant among *APOE* ε4 carriers, while there were no sex differences among *APOE* ε4 non-carriers. There were no sex differences in the amyloid-beta CSF biomarker.

Wang and colleagues (2018)^43^ showed no sex differences in amyloid-beta and tau CSF biomarkers, while females had more amyloid-beta pathology on the positron emission tomography (PET) biomarker. Zhang and colleagues (2018)^47^ showed more amyloid-beta pathology on PET.

Snitz and colleagues (2015)^40^ did not find any sex differences on amyloid-beta pathology on PET, in a cohort of cognitively unimpaired individuals spanning from no to several subjective cognitive complaints.

In summary, sex differences in AD biomarkers among individuals with SCD remain inconclusive, although some studies suggest that females, especially *APOE* ε4 carriers, may present with greater tau and amyloid pathology than men.

#### Basal forebrain resting-state functional connectivity (RSFC)

We did not find studies directly testing for the moderator effect of sex in the association between SCD and basal forebrain biomarkers. However, we found one study directly testing for the interaction between sex and PET-measured global amyloid levels, when predicting biomarkers of the cholinergic system, operationalized as RSFC of the basal forebrain in a cohort of 267 SCD individuals.^32^ The authors found a moderating effect of sex in the association between global amyloid levels and RSFC of the basal forebrain, revealing that higher amyloid deposits were more strongly associated with the basal forebrain functional connectivity in frontal and subcortical areas in SCD females compared to males. Thus, this study provides evidence on a moderating effect of sex in the impact of amyloid deposits over functional connectivity of the basal forebrain in SCD. Nevertheless, since the study lacks a control group without complaints, it is unclear whether the same effect is also present in non-SCD individuals.

#### Brain volume

We did not find any study directly testing for the moderating effect of sex on the association between SCD and brain volume. However, one study tested for differences between SCD males and SCD females in the volume of different brain areas.^43^ There were significantly larger volumes of hippocampus, entorhinal cortex, fusiform and medial temporal lobe in SCD males compared to females. Nevertheless, no correction for head size is mentioned and since the study lacks a control group without complaints, it is unclear whether the same effect is also present in non-SCD individuals.

#### Urinary cortisol

We found one study testing for the moderating effect of sex on the association between SCD and cortisol levels, using an interaction model between sex (i.e., males versus females) and SCD (i.e., presence/absence of SCD) in relation to urinary cortisol levels.^44^ The authors found that females with SCD showed significantly higher levels of cortisol compared to females without SCD. There were no differences found for the males. Thus, there is evidence of an effect of sex in the association of SCD with urinary cortisol levels.

#### Mental health

Vestberg and colleagues (2007)^42^ examined whether sex moderates the association between cognitive status, defined as objective memory impairment (OMI) versus subjective memory impairment (SMI), and depressive symptoms in a clinical sample of individuals with memory complaints. The authors found a significant interaction of sex and cognitive status (SMI versus OMI) and the levels of depressive symptomatology measured by Hospital Anxiety and Depression Scale (HADS). Specifically, among patients with SMI, females reported higher levels of depressive symptoms than males, whereas depressive symptom levels were similar across sexes in the OMI group (β = –4.9, p = 0.038, 95% CI: −9.59 to −0.27). This suggests that sex may influence how objective cognitive status relates to depressive symptoms among individuals presenting with memory complaints. Although this study did not include a control group without memory complaints, it provides evidence that sex may moderate the relationship between cognitive status and depressive symptomatology.

#### Mobility and balance

We did not find any study directly testing for the moderating effect of sex on the association between SCD and mobility and balance. However, one study^45^ tested sex-related differences between individuals with SCD and cognitively normal individuals. The authors found that females with SCD had significant impairment in mobility and balance compared to cognitively normal females (p < 0.001). In contrast, in males, there were no significant differences among groups for balance or mobility.

#### Sickness absence and disability benefits

We did not find any study directly testing for the moderating effect of sex on the association between SCD and sickness absence (SA) and disability benefits. Nonetheless, two studies using the same cohort^38,39^ evaluated sex differences on SA^38^ and disability benefits^39^ in SCD.

In the first study, Pihlajamäki and colleagues (2020)^38^ found that the odds ratio (OR) for being susceptible to any SA was 1.70 (1.35–2.13) for females and 1.45 (1.17–1.78) for males with SCD. The corresponding rate ratios (RR) for SA duration were 2.62 (2.06–3.33) for females and 3.25 (2.49–4.25) for males. Thus, SCD females showed qualitatively increased risk of SA but shorter periods of SA compared to males.

Regarding disability benefits, Pihlajamäki and colleagues (2021)^39^ found that SCD predicted disability benefits in both sexes when controlling for age and prior SA. Hazard ratios were 2.9 (95% CI = 1.4–6.0) for females and 3.7 (95% CI = 1.8–7.9) for males.

## Discussion

In this systematic review, we focused on the moderating effect of sex on the association between SCD and relevant health outcomes such as cognitive decline, dementia, and mortality. Overall, most of the included studies relied on sex groups comparisons rather than formal moderation models, limiting the extent to which true moderation can be assessed. However, the available literature indicates that females with SCD may show an elevated risk for future memory decline and dementia, while males with SCD may show increased risk of mortality. Moreover, the reviewed literature suggests sex-related differences in the levels of amyloid and tau biomarkers, urinary cortisol, brain volume and functional connectivity in individuals with SCD.

The interest in SCD stems mainly from its potential as one of the earliest symptoms of objective cognitive impairment and dementia.^1–3^ The evidence reviewed here supports the idea of SCD as an early marker of dementia both for males and females, but this risk might be especially relevant in females with SCD.^30,36,37^ Interestingly, objective cognitive performance in cross-sectional assessments did not demonstrate marked disparities,^33^ with females even exhibiting equal or superior cognitive performance.^43^ By definition, cognitive performance at baseline in SCD is comparable to that of individuals without cognitive complaints,^4^ explaining the divergent results. However, a steeper prospective decline in memory functions was evident in females over time,^34^ underscoring the nuanced nature of sex-related cognitive trajectories in the context of SCD. Nonetheless, these findings remain tentative given the limited number of studies that directly testing moderation analyses, reinforcing the necessity for well-powered longitudinal research to conclusively determine sex-specific risk patterns.

Males with SCD had a higher risk of mortality than females with SCD, especially when presented jointly with self-reported difficulties in activities of daily living.^30^ The contrast among SCD individuals regarding the risk of death (higher in males) and the risk of developing dementia (higher in females), may be explained by disparities in life expectancy.^48^ Longer life expectancy may provide females more time to develop cognitive decline and dementia compared to males. It is noteworthy that Strand and colleagues (2018)^41^ observed that males lived on average three years less than females in the follow-up period after the age of 75. Although activities of daily living are preserved in SCD, the association between subclinical difficulties and cognitive complaints has previously been reported in this population.^14,17^ Hence, we found that in SCD, complaints related to activities of daily living were associated with different patterns of outcomes in males and females, although these sex differences did not reach statistical significance. Thus, the evidence from the current systematic review supports personalized early interventions in SCD depending on sex. However, whether these sex-related differences result from a true moderating effect of sex in SCD or from broader population-level sex differences remains unclear, underscoring the importance of future investigations that include robust comparison groups without SCD.

Regarding biomarkers of underlying AD pathology, the evidence on the moderating effect of sex is limited due to the absence of studies comparing SCD with healthy controls without complaints. Building on previous findings showing an association between SCD and AD,^5,21,22^ the four studies reviewed did not test whether sex moderates the relationship between SCD and AD biomarkers. However, they provide mixed evidence of sex differences in biomarker levels, suggesting that this may be an important area for future research. Although SCD was previously associated with increased amyloid and tau levels,^21,22^ as well as lower cholinergic integrity,^49^ this association was similar for males and females. Interestingly, Babapour and colleagues (2020)^31^ showed that among *APOE ε*4 carriers, females exhibited significantly higher levels of tau biomarkers compared to males. Together with the sex-specific trajectories regarding dementia and death in SCD, the findings of Babapour and colleagues (2020)^31^ raise important questions about the interplay of genetic profiles, pathology, and sex in SCD. The moderating effect of sex was not studied in biomarkers of other pathologies such as cerebrovascular disease measured with white matter signal abnormalities (WMSA) on MRI.^6^ These WMSA have shown increased frequency in cognitively unimpaired females in the general population.^50^ Altogether, future research should investigate sex-specific pathology findings in SCD, taking into consideration the role of different genetic profiles and biomarkers beyond amyloid-beta and tau. Further, the potential moderating effect of sex on the association between SCD and AD biomarkers should be tested directly and using other independent cohorts in future studies.

Other outcome measures in this review included balance, sickness absence, urinary cortisol, and mental health. The evidence shows that females with SCD present with worse mobility and balance, as well as poorer mental health and increased levels of urinary cortisol as a proxy of stress levels.^42,44,45^ These findings are in line with the so-called sex paradox, where men show better general health compared to females but lower life expectancy.^48^ We cannot exclude that gender roles in our society may have impacted the reviewed studies on health outcomes. Gender roles and behaviors contribute to sex-based differences in health outcomes, with females being more prone to seek healthcare due to traditional family health roles.^12^ Traditional gender roles may incline females to report health issues more openly than males. Studies show females report more symptoms and take more days off work for health reasons,^13,51^ enabling the early detection of potentially life-threatening problems. In the current systematic review, these findings align with Pihlajamäki and colleagues (2020),^38^ where SCD females showed a higher risk of sickness absence but shorter absence periods than SCD males. Regular visits to health care services facilitate preventive interventions and early detection of disease.^52^ Skipping periodic health assessments might be associated with more severe presentations of disease and death. Whether the lower rates of help-seeking behaviors play a role in the longer sickness absence and increased risk of death in SCD males remains unanswered.

Certain methodological limitations must be considered in our review. Given the broad aim of the current study, a specific outcome measure was not determined a priori, which leads to substantial heterogeneity among the studies, hindering the inclusion of a meta-analysis. This wider approach provided a broader vision of the moderator effect of sex on many important health outcomes. Most of the included studies did not formally test for moderating effect (i.e., SCD by sex interactions), raising questions about whether the observed sex differences are comparable to those in control individuals without SCD. The specific role of sex and gender in SCD has been established as a priority by the international SCD initiative,^25^ and the current systematic review evidences a lack of studies focusing on that area. Indeed, this review's findings illustrate the need for methodologically rigorous studies that adopt clear moderation models and include appropriate control groups, to move beyond general sex group comparisons. To note, the variability among SCD operationalization methods and the samples included could be a source of heterogeneity across studies partially explaining some contradicting results. Although it was mandatory for all studies to fulfill the basic SCD-I criteria^4^ to be included in the present review, the extent to which studies applied additional ‘SCD-plus’ features (i.e., memory-specific complaints, onset within the last five years, concern associated with decline, and age >60) was not always reported. We did not identify studies that explicitly operationalized SCD + instead of SCD. Nevertheless, most studies included memory-related complaints and had a mean participant age above 60, which suggests that they would meet the SCD + risk enrichment profile. Some studies, however, did not report age distributions or the specific type of cognitive complaint used to classify SCD, which may have contributed to variability across findings. These issues highlight the importance of standardizing SCD assessment and reporting, as well as clarifying whether risk enrichment features are present, to improve comparability across studies.

Finally, we acknowledge the possibility that some relevant evidence may not have been identified through our search strategy. This could include studies published after our search end date or articles that were available during our search period but not indexed in the databases we consulted. As with any systematic review, the possibility of missing evidence cannot be fully excluded, and this should be considered when interpreting our findings. These limitations further emphasize the importance of regularly updating systematic reviews in rapidly evolving research areas such as SCD, to ensure that conclusions are based on the most comprehensive and up-to-date body of evidence. However, the current findings still cover a broad timeframe and evidences the underexplored moderating effect of sex in SCD. We acknowledge these limitations and their potential impact on the generalizability and robustness of the results obtained should be considered.

In conclusion, while our comprehensive overview suggests that sex may be a relevant factor in the association between SCD and several health outcomes, the moderating effect of sex has not been systematically tested in most studies. The most consistent evidence to date pertains to the potential moderating role of sex in the relationship between SCD and future objective cognitive decline. Although findings are mixed, some studies report stronger associations in females. These patterns underscore the importance of conducting more targeted research that explicitly examines sex as a moderating factor in SCD-related trajectories. These differential associations would enrich the development of sex-specific strategies focused on early intervention for AD. For example, strategies for females could focused on early cognitive stimulation and treatment for health outcomes, whereas strategies for males could be focused on increasing preventive visits to general practitioners, which could reduce their associated risk of death and sickness absence. However, a conclusion of this review is that the evidence available in the current literature is limited. These findings emphasize the need for an expanded evidence base, as further investigations are warranted to confirm and refine the role of sex as a moderator in SCD-related outcomes. Furthermore, the role of other potential variables that could be contributing to these divergent outcomes, such as age, education or genetic predispositions should also be considered in the future.

## Supplemental Material

sj-docx-1-alz-10.1177_13872877251397411 - Supplemental material for Underexplored moderating effects of sex in subjective cognitive decline: A systematic review and evidence gap Supplemental material, sj-docx-1-alz-10.1177_13872877251397411 for Underexplored moderating effects of sex in subjective cognitive decline: A systematic review and evidence gap by Yolanda Álvarez-Pérez, Andrea Duarte-Díaz, Yaiza Molina, Nerea Figueroa-Lamas, Patricia Diaz-Galvan, Eloy García-Cabello, Lissett Gonzalez-Burgos, Roraima YÁnes-Pérez, Amado Rivero-Santana, Jonas K Olofsson, Jose Barroso, Daniel Ferreira, Lilisbeth Perestelo-Pérez and Nira Cedres in Journal of Alzheimer's Disease

## References

[1] Jessen F Amariglio RE Buckley RF , et al. The characterisation of subjective cognitive decline. Lancet Neurol 2020; 19: 271–278. 31958406 10.1016/S1474-4422(19)30368-0 PMC7062546

[2] Mitchell AJ Beaumont H Ferguson D , et al. Risk of dementia and mild cognitive impairment in older people with subjective memory complaints: meta-analysis. Acta Psychiatr Scand 2014; 130: 439–451. 25219393 10.1111/acps.12336

[3] Slot RER Sikkes SAM Berkhof J , et al. Subjective cognitive decline and rates of incident Alzheimer’s disease and non–Alzheimer’s disease dementia. Alzheimers Dement 2019; 15: 465–476. 30555032 10.1016/j.jalz.2018.10.003 PMC6465066

[4] Jessen F Amariglio RE van Boxtel M , et al. A conceptual framework for research on subjective cognitive decline in preclinical Alzheimer’s disease. Alzheimers Dement J Alzheimers Assoc 2014; 10: 844–852. 10.1016/j.jalz.2014.01.001 PMC4317324 24798886

[5] Rostamzadeh A Bohr L Wagner M , et al. Progression of subjective cognitive decline to MCI or dementia in relation to biomarkers for Alzheimer disease: a meta-analysis. Neurology 2022; 99: e1866–e1874. 10.1212/WNL.0000000000201072 36028322

[6] Pitti H Diaz-Galvan P Barroso J , et al. Cerebrovascular damage in subjective cognitive decline: a systematic review and meta-analysis. Ageing Res Rev 2022; 82: 101757. 36240992 10.1016/j.arr.2022.101757

[7] Castro-Aldrete L Moser MV Putignano G , et al. Sex and gender considerations in Alzheimer’s disease: the Women’s Brain Project contribution. Front Aging Neurosci 2023; 15: 1105620. 37065460 10.3389/fnagi.2023.1105620 PMC10097993

[8] Dong C Zhou C Fu C , et al. Sex differences in the association between cardiovascular diseases and dementia subtypes: a prospective analysis of 464,616 UK biobank participants. Biol Sex Differ 2022; 13: 21. 35526028 10.1186/s13293-022-00431-5 PMC9080133

[9] Liew TM . Subjective cognitive decline, APOE e4 allele, and the risk of neurocognitive disorders: age- and sex-stratified cohort study. Aust N Z J Psychiatry 2022; 56: 1664–1675. 35229693 10.1177/00048674221079217 PMC9433458

[10] Mosconi L Berti V Guyara-Quinn C , et al. Perimenopause and emergence of an Alzheimer’s bioenergetic phenotype in brain and periphery. PLoS One 2017; 12: e0185926. 10.1371/journal.pone.0185926 PMC5634623 29016679

[11] Mosconi L Rahman A Diaz I , et al. Increased Alzheimer’s risk during the menopause transition: a 3-year longitudinal brain imaging study. PLoS One 2018; 13: e0207885. 10.1371/journal.pone.0207885 PMC6291073 30540774

[12] Nagai S . Does male gender role conflict inhibit help-seeking? Jpn Psychol Res 2024; 66: 359–368.

[13] Green CA Pope CR . Gender, psychosocial factors and the use of medical services: a longitudinal analysis. Soc Sci Med 1999; 48: 1363–1372. 10369437 10.1016/s0277-9536(98)00440-7

[14] Cedres N Machado A Molina Y , et al. Subjective cognitive decline below and above the age of 60: a multivariate study on neuroimaging, cognitive, clinical, and demographic measures. J Alzheimers Dis 2019; 68: 295–309. 30741680 10.3233/JAD-180720

[15] Harada CN Natelson Love MC Triebel KL . Normal cognitive aging. Clin Geriatr Med 2013; 29: 737–752. 24094294 10.1016/j.cger.2013.07.002 PMC4015335

[16] Hoogendam YY Hofman A Van Der Geest JN , et al. Patterns of cognitive function in aging: the Rotterdam Study. Eur J Epidemiol 2014; 29: 133–140. 24553905 10.1007/s10654-014-9885-4

[17] Diaz-Galvan P Cedres N Figueroa N , et al. Cerebrovascular disease and depressive symptomatology in individuals with subjective cognitive decline: a community-based study. Front Aging Neurosci 2021; 13: 656990. 34385912 10.3389/fnagi.2021.656990 PMC8353130

[18] Ginó S Mendes T Maroco J , et al. Memory complaints are frequent but qualitatively different in young and elderly healthy people. Gerontology 2010; 56: 272–277. 19776545 10.1159/000240048

[19] Zlatar ZZ Moore RC Palmer BW , et al. Cognitive complaints correlate with depression rather than concurrent objective cognitive impairment in the successful aging evaluation baseline sample. J Geriatr Psychiatry Neurol 2014; 27: 181–187. 24614203 10.1177/0891988714524628 PMC4255945

[20] Zapater-Fajarí M Diaz-Galvan P Cedres N , et al. Biomarkers of Alzheimer’s disease and cerebrovascular disease in relation to depressive symptomatology in individuals with subjective cognitive decline. J Gerontol Ser A 2024; 79: glad216. 10.1093/gerona/glad216 PMC10803123 37708068

[21] Amariglio RE Becker JA Carmasin J , et al. Subjective cognitive complaints and amyloid burden in cognitively normal older individuals. Neuropsychologia 2012; 50: 2880–2886. 22940426 10.1016/j.neuropsychologia.2012.08.011 PMC3473106

[22] Buckley RF Hanseeuw B Schultz AP , et al. Region-specific association of subjective cognitive decline with tauopathy independent of global β-amyloid burden. JAMA Neurol 2017; 74: 1455. 28973551 10.1001/jamaneurol.2017.2216 PMC5774633

[23] Sun M Zhang Q Han Y , et al. Sleep quality and subjective cognitive decline among older adults: the mediating role of anxiety/depression and worries. J Aging Res 2024; 2024: 4946303. 38746043 10.1155/2024/4946303 PMC11093690

[24] Zapater-Fajarí M Crespo-Sanmiguel I Perez V , et al. Subjective memory complaints in young people: the role of resilience. Psychol Health 2024; 39: 1243–1262. 36368933 10.1080/08870446.2022.2141240

[25] Munro CE Boyle R Chen X , et al. Recent contributions to the field of subjective cognitive decline in aging: a literature review. Alzheimers Dement (Amst) 2023; 15: e12475. 10.1002/dad2.12475 PMC10585124 37869044

[26] Page MJ McKenzie JE Bossuyt PM , et al. The PRISMA 2020 statement: an updated guideline for reporting systematic reviews. Br Med J 2021; 372: n71. 10.1136/bmj.n71 PMC8005924 33782057

[27] Clayton JA Tannenbaum C . Reporting sex, gender, or both in clinical research? JAMA 2016; 316: 1863. 27802482 10.1001/jama.2016.16405

[28] Heidari S Babor TF De Castro P , et al. Sex and gender equity in research: rationale for the SAGER guidelines and recommended use. Res Integr Peer Rev 2016; 1: 2. 29451543 10.1186/s41073-016-0007-6 PMC5793986

[29] Moola S Munn Z Tufanaru C , et al. Chapter 7: Systematic reviews of etiology and risk. In: Aromataris E Lockwood C Porritt K , et al. (eds) JBI Manual for Evidence Synthesis. Adelaide, South Australia: JBI, 2020, pp.298.

[30] Abdulrahman H Richard E van Gool WA , et al. Sex differences in the relation between subjective memory complaints, impairments in instrumental activities of daily living, and risk of dementia. J Alzheimers Dis 2022; 85: 283–294. 34806609 10.3233/JAD-215191 PMC8842768

[31] Babapour Mofrad R Tijms BM Scheltens P , et al. Sex differences in CSF biomarkers vary by Alzheimer disease stage and *APOE* ε4 genotype. Neurology 2020; 95: e2378–e2388. 10.1212/WNL.0000000000010629 32788242

[32] Chiesa PA Cavedo E Grothe MJ , et al. Relationship between basal forebrain resting-state functional connectivity and brain amyloid-β deposition in cognitively intact older adults with subjective memory complaints. Radiology 2019; 290: 167–176. 30351255 10.1148/radiol.2018180268

[33] Cipolli C Neri M Andermarcher E , et al. Self-rating and objective memory testing of normal and depressed elderly. Aging Clin Exp Res 1990; 2: 39–48. 10.1007/BF03323893 2094354

[34] Drouin SM McFall GP Dixon RA . In multiple facets of subjective memory decline sex moderates memory predictions. Alzheimers Dement (Amst) 2020; 12: e12089. 10.1002/dad2.12089 PMC7447903 32875056

[35] Heser K Kleineidam L Wiese B , et al. Subjective cognitive decline may be a stronger predictor of incident dementia in women than in men. J Alzheimers Dis 2019; 68: 1469–1478. 30909220 10.3233/JAD-180981

[36] Kryscio RJ Abner EL Cooper GE , et al. Self-reported memory complaints: implications from a longitudinal cohort with autopsies. Neurology 2014; 83: 1359–1365. 25253756 10.1212/WNL.0000000000000856 PMC4189103

[37] Pérès K Helmer C Amieva H , et al. Gender differences in the prodromal signs of dementia: memory complaint and IADL-restriction. a prospective population-based cohort. J Alzheimers Dis 2011; 27: 39–47. 21725162 10.3233/JAD-2011-110428

[38] Pihlajamäki M Arola H Ahveninen H , et al. Subjective cognitive complaints and sickness absence: a prospective cohort study of 7059 employees in primarily knowledge-intensive occupations. Prev Med Rep 2020; 19: 101103. 32420012 10.1016/j.pmedr.2020.101103 PMC7218151

[39] Pihlajamäki M Arola H Ahveninen H , et al. Subjective cognitive complaints and permanent work disability: a prospective cohort study. Int Arch Occup Environ Health 2021; 94: 901–910. 33462663 10.1007/s00420-020-01643-1 PMC8238735

[40] Snitz BE Weissfeld LA Cohen AD , et al. Subjective cognitive complaints, personality and brain amyloid-beta in cognitively normal older adults. Am J Geriatr Psychiatry 2015; 23: 985–993. 25746485 10.1016/j.jagp.2015.01.008 PMC4532656

[41] Strand BH Knapskog A-B Persson K , et al. Survival and years of life lost in various aetiologies of dementia, mild cognitive impairment (MCI) and subjective cognitive decline (SCD) in Norway. PLoS One 2018; 13: e0204436. 10.1371/journal.pone.0204436 PMC6150521 30240425

[42] Vestberg S Passant U Risberg J , et al. Personality characteristics and affective status related to cognitive test performance and gender in patients with memory complaints. J Int Neuropsychol Soc 2007; 13: 911–919. 17942009 10.1017/S1355617707071159

[43] Wang L Tian T ; Alzheimer’s Disease Neuroimaging Initiative. Gender differences in elderly with subjective cognitive decline. Front Aging Neurosci 2018; 10: 166. 29915534 10.3389/fnagi.2018.00166 PMC5994539

[44] Wolf OT Dziobek I McHugh P , et al. Subjective memory complaints in aging are associated with elevated cortisol levels. Neurobiol Aging 2005; 26: 1357–1363. 16243606 10.1016/j.neurobiolaging.2004.11.003

[45] Yoon B Choi SH Jeong JH , et al. Balance and mobility performance along the Alzheimer’s disease spectrum. J Alzheimers Dis 2020; 73: 633–644. 31815691 10.3233/JAD-190601

[46] Oliver MD Morrison C Kamal F , et al. Subjective cognitive decline is a better marker for future cognitive decline in females than in males. Alzheimers Res Ther 2022; 14: 197. 36581949 10.1186/s13195-022-01138-w PMC9798694

[47] Zhang J Zhou W Cassidy RM , et al. Risk factors for amyloid positivity in older people reporting significant memory concern. Compr Psychiatry 2018; 80: 126–131. 29091778 10.1016/j.comppsych.2017.09.015

[48] Oksuzyan A Juel K Vaupel JW , et al. Men: good health and high mortality. Sex differences in health and aging. Aging Clin Exp Res 2008; 20: 91–102. 18431075 10.1007/bf03324754 PMC3629373

[49] Rodriguez-Hernandez MA Alemany I Olofsson JK , et al. Degeneration of the cholinergic system in individuals with subjective cognitive decline: a systematic review. Neurosci Biobehav Rev 2024; 157: 105534. 38220033 10.1016/j.neubiorev.2024.105534

[50] Graff-Radford J Aakre JA Knopman DS , et al. Prevalence and heterogeneity of cerebrovascular disease imaging lesions. Mayo Clin Proc 2020; 95: 1195–1205. 32498775 10.1016/j.mayocp.2020.01.028 PMC7316133

[51] Ladwig KH Marten-Mittag B Formanek B , et al. Gender differences of symptom reporting and medical health care utilization in the German population. Eur J Epidemiol 2000; 16: 511–518. 11049093 10.1023/a:1007629920752

[52] McIsaac WJ Fuller-Thomson E Talbot Y . Does having regular care by a family physician improve preventive care? Can Fam Physician Med Fam Can 2001; 47: 70–76. PMC2014713 11212436

