# Supplementary material for: Underexplored moderating effects of sex in subjective cognitive decline: A systematic review and evidence gap
Source: J Alzheimers Dis. 2025 Nov 28;109(2):604–18. doi: 10.1177/13872877251397411 (PMC12775175; doi:10.1177/13872877251397411)
Supplement: sj-docx-1-alz-10.1177_13872877251397411 - Supplemental material for Underexplored moderating effects of sex in subjective cognitive decline: A systematic review and evidence gap [file sj-docx-1-alz-10.1177_13872877251397411.docx]

**Supplemental Material**

**Underexplored moderating effects of sex in subjective cognitive decline: A systematic review and evidence gap**

| **Medline** |
| --- |
| exp Cognitive Reserve/ |
| (subjective cognitive adj (decline or complaint$)).ti,ab,kw. |
| ((cognitive or memory) adj1 complaint*).ti,ab. |
| ("subjective memory impairment" or "subjective cognitive impairment" or "subjective memory decline").ti,ab. |
| 1 or 2 or 3 or 4 |
| *Aging/ or Aged/ |
| Middle Aged/ |
| Age Factors/ |
| Cognition/ |
| Memory/ |
| Brain/ |
| Executive Function/ |
| (aging or aged or "middle age*" or age* factor$ or cognition or memor? or brain or "executive function" or perform* or "mental perform*").ti,ab. |
| AAMI.ti,ab. |
| ACMI.ti,ab. |
| ARCD.ti,ab. |
| 6 or 7 or 8 or 9 or 10 or 11 or 12 or 13 or 14 or 15 or 16 |
| 5 and 17 |
| (gender$ or sex$).af. |
| (boys or girls).tw. |
| (women or men).ti. |
| (male$1 or female$1).ti. |
| (women or men).ab. /freq=4 |
| (male$1 or female$1).ab. /freq=4 |
| (women adj8 men).ab. |
| (female$1 adj8 male$1).ab. |
| 19 or 20 or 21 or 22 or 23 or 24 or 25 or 26 |
| 18 and 27 |
| exp Educational Measurement/ |
| exp Educational Status/ |
| (education or "years of education" or "educational attainment" or "educational measurement" or "educational status").ti,ab. |
| 29 or 30 or 31 |
| 18 and 32 |

| **Cochrane** |
| --- |
| MeSH descriptor: [Cognitive Reserve] explode all trees |
| (subjective cognitive adj1 (decline or complaint*)) |
| ((cognitive or memory) adj1 complaint*) |
| ("subjective memory impairment" or "subjective cognitive impairment" or "subjective memory decline") |
| #1 or #2 or #3 or #4 |
| MeSH descriptor: [Aged] explode all trees |
| MeSH descriptor: [Middle Aged] explode all trees |
| MeSH descriptor: [Age Factors] explode all trees |
| MeSH descriptor: [Cognition] explode all trees |
| MeSH descriptor: [Memory] explode all trees |
| MeSH descriptor: [Brain] explode all trees |
| MeSH descriptor: [Executive Function] explode all trees |
| (aging or aged or "middle age*" or age* factor* or cognition or memor* or brain or "executive function" or perform* or "mental perform*") |
| AAMI |
| ACMI |
| ARCD |
| #6 or #7 or #8 or #9 or #10 or #11 or #12 or #13 or #14 or #15 or #16 |
| #5 AND #17 |
| MeSH descriptor: [Educational Measurement] explode all trees |
| MeSH descriptor: [Educational Status] explode all trees |
| (education or "years of education" or "educational attainment" or "educational measurement" or "educational status") |
| #19 OR #20 OR #21 |
| #18 AND #22 |
| (gender or sex) |
| (male or female) |
| (boys or girls) |
| (women or men) |
| #24 or #25 or #26 or #27 |
| #18 and #28 |

| **WOS** |
| --- |
| TS= ((subjective cognitive NEAR/1 (decline or complaint*))) |
| TS= ((cognitive or memory) near/1 complaint*) |
| TS= ("subjective memory impairment" or "subjective cognitive impairment" or "subjective memory decline") |
| #3  OR #2  OR #1 |
| TS= (aging or aged or "middle age*" or age* factor* or cognition or memory or brain or "executive function" or "mental perform*") |
| TS= (AAMI  OR ACMI  OR ARCD) |
| #6  OR #5 |
| #7  AND #4 |
| TS= (education or "years of education" or "educational attainment" or "educational measurement" or "educational status") |
| #9  AND #8 |
| TS= (boys or girls) |
| TI= (male or female) |
| TI= (women or men) |
| TS= (gender or sex) |
| #14  OR #13  OR #12  OR #11 |
| #15  AND #8 |

| **CINHAL** |
| --- |
| TI cognitive reserve OR AB cognitive reserve |
| TI ( (subjective cognitive N1 (decline or complaint*) ) OR AB ( (subjective cognitive N1 (decline or complaint*) ) |
| TI ( ((cognitive or memory) N1 complaint*) ) OR AB ( ((cognitive or memory) N1 complaint*) ) |
| TI("subjective memory impairment" or "subjective cognitive impairment" or "subjective memory decline") or AB ("subjective memory impairment" or "subjective cognitive impairment" or "subjective memory decline") |
| S1 OR S2 OR S3 OR S4 |
| MH Aged OR MH Aging |
| MH Age factors |
| MH cognition |
| MH memory |
| MH brain |
| MH Executive Function |
| TI ( (aging or aged or "middle age*" or "age* factor*" or cognition or memor* or brain or "executive function" or perform* or "mental perform*") ) OR AB ( (aging or aged or "middle age*" or "age* factor*" or cognition or memor* or brain or "executive function" or perform* or "mental perform*") ) |
| TI AAMI OR AB AAMI |
| TI ACMI OR AB ACMI |
| TI ARCD OR AB ARCD |
| S6 OR S7 OR S8 OR S9 OR S10 OR S11 OR S12 OR S13 OR S14 OR S15 |
| S5 AND S16 |
| (gender or sex) |
| TX (boys or girls) |
| TI (women or men) |
| TI (male or female) |
| AB (male or female) |
| AB (women or men) |
| AB (women N8 men) |
| AB (female N8 male) |
| (S18 OR S19 OR S20 OR S21 OR S22 OR S23 OR S24 OR S25) |
| (S17 AND S26) |
| (MH "Educational Measurement+") |
| (MH "Educational Status") |
| TI (education or "years of education" or "educational attainment" or "educational measurement" or "educational status") OR AB (education or "years of education" or "educational attainment" or "educational measurement" or "educational status") |
| S28 OR S29 OR S30 |
| S17 AND S31 |
